# Incorporating Novel Technologies in Precision Oncology for Colorectal Cancer: Advancing Personalized Medicine

**DOI:** 10.3390/cancers16030480

**Published:** 2024-01-23

**Authors:** Pankaj Ahluwalia, Kalyani Ballur, Tiffanie Leeman, Ashutosh Vashisht, Harmanpreet Singh, Nivin Omar, Ashis K. Mondal, Kumar Vaibhav, Babak Baban, Ravindra Kolhe

**Affiliations:** 1 Department of Pathology, Medical College of Georgia at Augusta University, Augusta, GA 30912, USA; pahluwalia@augusta.edu (P.A.); kballur@augusta.edu (K.B.); tleeman@augusta.edu (T.L.); avashisht@augusta.edu (A.V.); hsingh1@augusta.edu (H.S.); nomar@augusta.edu (N.O.); amondal@augusta.edu (A.K.M.); 2 Department of Neurosurgery, Augusta University, Augusta, GA 30912, USA; kvaibhav@augusta.edu; 3 Departments of Neurology and Surgery, Augusta University, Augusta, GA 30912, USA; bbaban@augusta.edu

**Keywords:** predictive, preventive, personalized, equitable medicine, colorectal cancer, gene signature, spatial, clinical, genomics, prognostic, immune infiltration, precision medicine, stratified medicine, immunotherapy response

## Abstract

**Simple Summary:**

Cancer affects millions of individuals every year, with colorectal cancer being among the most common. There is an increased need to identify new biomarkers that can not only diagnose patients early, but also stratify them so the best treatment can be initiated for each patient. Every human has a unique genetic makeup that causes them to respond differently to cancer. In recent years, new technologies have provided unprecedented access to tumor samples from patients. Through these analyses, we can not only diagnose and classify patients based on their comparative risk, but also monitor their response to emerging therapies. Continued progress using these methods will transform how we approach treatment modalities for cancer patients.

**Abstract:**

Colorectal cancer (CRC) is one of the most heterogeneous and deadly diseases, with a global incidence of 1.5 million cases per year. Genomics has revolutionized the clinical management of CRC by enabling comprehensive molecular profiling of cancer. However, a deeper understanding of the molecular factors is needed to identify new prognostic and predictive markers that can assist in designing more effective therapeutic regimens for the improved management of CRC. Recent breakthroughs in single-cell analysis have identified new cell subtypes that play a critical role in tumor progression and could serve as potential therapeutic targets. Spatial analysis of the transcriptome and proteome holds the key to unlocking pathogenic cellular interactions, while liquid biopsy profiling of molecular variables from serum holds great potential for monitoring therapy resistance. Furthermore, gene expression signatures from various pathways have emerged as promising prognostic indicators in colorectal cancer and have the potential to enhance the development of equitable medicine. The advancement of these technologies for identifying new markers, particularly in the domain of predictive and personalized medicine, has the potential to improve the management of patients with CRC. Further investigations utilizing similar methods could uncover molecular subtypes specific to emerging therapies, potentially strengthening the development of personalized medicine for CRC patients.

## 1. Colorectal Cancer—Carcinogenesis and Clinical Management

Colorectal cancer is one of the deadliest forms of cancer, with approximately 1.5 million new cases diagnosed annually [1]. The 5-year survival rate for localized disease is 90%, but this drastically decreases to 14% with the distant-stage disease [2]. Additionally, socio-economic status, age, and African-American ethnicity have been linked as risk factors for CRC [3]. Further, variability due to sexual differences, genetic heterogeneity, and underlying causes makes the clinical management of CRC complex [4]. A deeper understanding of the molecular biology of cancer and novel approaches to translate them into clinical applications would greatly help in the management of CRC. In this review article, we began with the basics of cancer and then discussed the clinical management of colorectal cancer (CRC). We then discussed the potential of several breakthrough translational studies that can help identify and understand molecular signatures with prognostic and predictive value. Emerging approaches that show promise are discussed, including gene expression signatures, liquid biopsy, single-cell sequencing, and spatial transcriptomics. As these technologies continue to improve, their integration using the principles of personalized medicine could significantly benefit CRC patients.

## 2. Introduction

The development and progression of cancer are the results of accumulated mutations and functional perturbations in the cancer cell, which give it a survival advantage, higher proliferation, and the ability to evade the host’s immune system. Over the years, several molecular hallmarks have been identified [5,6]. The central characteristic of cancer cells is their property to sustain proliferation. This is achieved through cellular signaling, which promotes cell cycle progression and metabolism [7]. Cancer cells can produce growth factor ligands, send signals to normal cells to activate growth factors, and thus lead to growth. Mutations can also activate pathways that would normally be triggered by growth factor receptors, such as BRAF protein activating the MAPK pathway or the PI3K pathway. Multiple genes, such as EGFR, RAS, RAF, TGFBR2, TGFBR1, SMADs, AXIN, and CTNNB1, have been identified as playing a significant role in the proliferation, progression, and invasion of cancer cells [8,9]. One of the most interesting genes, TGF-β (Transforming Growth Factor- β) plays a paradoxical role in CRC progression [10]. In normal tissue, TGF-β inhibits the proliferation of intestinal epithelia and promotes apoptosis. TGF-β acts as a tumor suppressor in these conditions. However, during tumorigenesis, CRC tumors lose these suppressor properties. In the absence of these suppressor proteins, these tumors are resistant to TGF-β-induced growth inhibition [11,12,13]. In the late stages of colorectal cancer (CRC) tumors, TGF-β is highly expressed and acts as a tumor promoter, as the production of several growth factors such as Transforming Growth Factor-α (TGF-α), Fibroblast Growth Factor (FGF) and Epidermal Growth Factor (EGF) is increased. [10].

Cancer cells lack contact inhibition, whereby there is decreased cell proliferation as cell density increases [14]. The Hippo pathway is evolutionarily conserved and plays a critical role in regulating tissue growth. Two crucial transcription co-activators, YAP, and TAZ, operate downstream and mediate the main gene regulation and biological activities of the Hippo pathway [15]. Dysregulation of the Hippo pathway or overexpression of YAP can cause cells to break through the barrier of contact inhibition [16]. Several other mechanisms regulate gene expression, including microRNA. Recently, gene regulation, such as microRNA-based regulation, has played a central role in CRC carcinogenesis. For example, the DICER1-miR-590-5p-YAP1 axis has emerged as dysregulated in colorectal tumorigenesis [17]. Other emerging hallmarks include phenotypic plasticity, epigenetic reprogramming, the role of microbiomes, and senescent cells [18].

Clinically, the American Joint Committee on Cancer (AJCC)/Union for International Cancer Control (UICC)/Tumor Node Metastasis (TNM) classification system has been the fundamental tool for assessing prognosis and determining treatment options for solid tumors [19]. TNM staging, along with genomic profiling, is important for the clinical management of CRC. Patients with stage I or II tumors have a better prognosis than those with higher-stage III or IV cancers. Additionally, there is variability in survival prediction for stage II patients, with a 5-year survival rate of 73, while stage III patients have a survival rate of 55% [20]. The TNM staging system is regularly updated with new information, such as vascular invasion, tumor budding, and molecular features such as BRAF and mismatch repair (MMR) status. Nevertheless, the well-known “stage paradox” of colon cancer, in which the prognosis is better for patients at the later stage of IIIA than at the earlier stage of IIB/IIC, has been a persistent observation throughout different versions of the TNM system. Despite this, a “stage paradox” has been observed in colorectal cancer (CRC) patients, in which patients in stage IIIA have improved outcomes compared to those in stage IIB/IIC [21,22]. Several studies have investigated the molecular mechanisms underlying this paradox. For instance, lysophospholipid metabolic pathways and MRAS-MAPK pathways were enriched in stage IIB/IIC CRC patients, which has been linked to the aggressiveness of cancer in this subgroup [23,24,25].

The treatment of colorectal cancer usually involves surgery and adjuvant therapy. Chemotherapy regimens include drugs such as fluoropyrimidine (5-FU), irinotecan (IRI), oxaliplatin (OX), and capecitabine, both as single agents and in combination, based on the clinical presentation of the disease [26,27,28,29]. Patients with advanced stage and proficient mismatch repair proteins (MMR) are eligible for adjuvant therapy [30]. Patients with colorectal cancer (CRC) in high-risk stages II and III are recommended to receive combined fluoropyrimidine and oxaliplatin chemotherapy to improve overall survival [31]. EGFR has been known to play a central role in tumor progression for almost three decades. This has led to advancements in targeted therapies against EGFR [32,33]. Cetuximab and panitumumab (anti-EGFR) antibodies have shown improved overall survival of greater than three months in metastatic CRC [34,35]. Anti-EGFR therapy is recommended based on the mutational status of several critical genes such as BRAF (V600E), PIK3CA, and KRAS (exon 2) in metastatic colorectal cancer [36]. The mutation in exon 2 of the KRAS gene was found in up to 45% of patients with metastatic cancer, activating the MAPK signaling pathway and rendering anti-EGFR blockade ineffective [37,38]. Only patients with tumors containing the normal KRAS gene benefited from EGFR inhibition. Additionally, other markers associated with a lack of response were identified, such as molecular alterations in NRAS, BRAF, PTEN, and PIK3CA [39].

Although these therapies have markedly improved outcomes in some patients, the lack of robust biomarkers has led to the problem of insufficient or over-treatment of a significant proportion of patients [40,41,42]. Approximately 60-80% of individuals with stage II CRC can be successfully treated with curative surgery, and only 4% of these patients are thought to benefit from adjuvant chemotherapy [43]. To ensure that patients are not overtreated, prognostic classification is necessary to differentiate between those at higher risk of mortality and those at lower risk, regardless of treatments.

## 3. The Clinical Relevance of Molecular Features of Colorectal Cancer

In 2015, an international consortium developed a consensus molecular classification system that divided CRC patients into four subtypes (CMS1 to CMS4) [44]. The CMS1 subtype is characterized by immune activation, microsatellite instability (MSI), hypermutation, CpG island methylator phenotype (CIMP), and BRAF mutation, and makes up 14% of early-stage tumors. CMS2 subtype is canonical, epithelial, activated WNT, and MYC signaling, as well as chromosomal instability (CIN), which make up to 37% of early-stage tumors. CMS3 subtype with metabolic dysregulation, epithelial characteristics, and KRAS mutation make up 13% of tumors. CMS3 is characterized by dysregulation of metabolic pathways, including carbohydrate and fatty acid oxidation, and a loss of TH17 cells. The last subtype, CMS4, has mesenchymal characteristics, epithelial-mesenchymal transition, stromal invasion, and angiogenesis, making up 23% of early-stage tumors. CMS4, on the other hand, is characterized by elevated matrix remodeling, complement activation, stromal infiltration, platelet activation, and immune upregulation. The remaining 13% of patients showed mixed phenotype characteristics of intratumoral heterogeneity [44,45]. 

The patient stratification based on CMS subtypes can provide potentially prognostic information, as CMS1 patients with an enriched immune microenvironment have a better prognosis compared to CMS4 subtypes, which are enriched in fibroblasts [46]. The main strengths of the system have been its stage-independent prognostic capabilities and its ability to identify poor outcomes for relapse-free and overall survival in CMS4 [44]. The CMS subtype system has shown promising potential for predicting the effectiveness of chemotherapy for advanced metastatic colorectal cancer, although validation of the results is limited [47]. However, patient classification using this signature does not take into account stromal-derived intra-tumoral heterogeneity (ITH), which can reduce the accuracy of predictions or prognoses. Further, the need for sufficient tumor material, as well as the cost and technology needed for expression analysis, limits its widespread applicability.

In 2017, the Colorectal Cancer Intrinsic Subtypes (CRIS) system was developed, leveraging patient-derived xenografts (PDX) models. The PDX model has a stromal component of the mouse, compared to the original tumor. This has been advantageous, as it only captures gene expression variations in cancer cells, irrespective of surrounding stroma, and can be used for prognostic and predictive purposes [48]. Compared to the CMS classification system that focuses on gene expression of bulk tumor tissue containing both tumor and normal cells, CRIS offers a more refined classification system. CRIS specifically prioritizes epithelial-specific genes and extracts tumor intrinsic gene signature that exhibits improved prognostic power compared to the CMS subtype. The CRIS classification system can be stratified into five classes. CRIS-A showed mucinous, glycolytic, KRAS mutation or microsatellite instability (MSI) properties. CRIS-B showed higher expression of EMT and TGF-β activation and generally had a poorer prognosis. CRIS-C showed higher EGFR signaling and was sensitive to EGFR inhibitors. The CRIS-D subtype demonstrated WNT activation and amplification of the IGF2 gene. The CRIS-E subtype exhibited a Paneth cell-like phenotype, along with a TP53 mutation, in this subgroup. By limiting confounding factors associated with host stromal components, CRIS subtypes can successfully stratify distinct groupings of primary and metastatic colorectal cancers (CRCs), thus reducing stromal-derived intratumoral heterogeneity [48,49]. Among these subtypes, patients with the CRIS-C subtype showed lower infiltration of CD8 T cells. Almost 50% of CMS2 phenotype CRC patients can be attributed to CRIS-C. Patients with the CRIS-C subtype and an epithelial-rich CMS2 phenotype had better prognoses with surgery plus adjuvant chemotherapy compared to surgery alone in stages II and III. Further, the risk of relapse was found to be high in CRIS-C patients with low levels of CD8+ T cells in stage II and III CRC cancer. (H.R = 12.18, 95% C.I = 1.51–98.58; *p* = 0.03) [50]. Although these molecular classification tools have assisted in the identification of novel subtypes of CRC patients, their widespread utility is still limited. Additionally, there is currently no recommendation in international guidelines to employ these classifications for adjuvant therapy [31]. Thus, there is a pressing need to explore novel approaches and employ emerging technologies that can assist in the current treatment regimens of CRC patients.

## 4. Transcriptomics and Its Integration with Personalized Medicine

Traditionally, clinical tools such as tumor staging system TNM, microsatellite instability (MSI), and more recently, tumor mutation burden (TMB) have emerged as important markers for the management of cancer patients [51]. However, these markers have limitations, lack accuracy, and do not fully capture the tumor heterogeneity [52,53]. While molecular profiling of tumors and cataloging of genetic mutations have emerged as crucial tools in the clinic, there are still limitations preventing the full realization of its potential in prognosis and prediction [54]. Transcriptomics has emerged as a powerful tool in improving the identification of prognostic signatures in colorectal cancer (CRC). Transcriptional signatures, including panel-based gene expression assays such as Oncotype Dx, ColoPrint, and GeneFx, have shown promise in improving upon traditional methods, but they need further development and require additional validations [55,56,57]. To fill in the gap in accurate and effective biomarkers in colorectal cancer, the development of robust algorithms for gene expression and molecular classification is essential for achieving more accurate and personalized cancer management. 

There have been several gene signatures that have been identified for their prognostic significance in colorectal cancer. Over the last decade, multiple signatures have identified the prognostic role of mRNA, miRNA, or lncRNA in colorectal cancer [58,59] (Table 1). Although several studies on single-gene-based prognostic markers have been reported, multiple-gene prognostic biomarkers have been found to provide an improved prognostic classification of CRC patients [60]. To address the gap in accurate and effective biomarkers, it is essential to develop robust algorithms including novel molecular classification systems that utilize gene expression to achieve more precise and personalized management in colorectal cancer. Several studies have identified novel molecular transcription-based profiles, which have greatly helped in better understanding the underlying heterogeneity of this complex disease. These diverse pathways, including metabolism, immune function, and cell interaction-based gene signatures, have enhanced our understanding of CRC, and provided new insights into potential prognostic indicators. In addition to transcriptomics, advancements in spatial biology, single-cell analysis, and proteomics have explained an additional layer of complexity associated with cancer progression, thus enabling the identification of new molecular subtypes and potential therapeutic targets. This multidisciplinary approach holds immense potential in advancing personalized treatment options for colorectal cancer patients. The paradigm of personalized medicine is an integrative approach to healthcare that aims to develop interdisciplinary research and management in the field of healthcare to augment comprehensive disease monitoring, leading to improved outcomes for patients [61,62]. Using these approaches, the conventional TNM classification system can incorporate emerging technologies in the clinical framework. The quantification of genes using a custom gene panel and its assessment at multidimensional levels has identified its prognostic, predictive, and personalized utility, which has the additional benefit of being cost-effective and further strengthening equitable medicine. Further, the utilization of single-cell technologies and identification of the spatial distribution of therapeutically relevant biomolecules can provide a high-resolution view of molecular alterations.

## 5. Emerging Insights from Single-Cell Analyses in CRC

Bulk transcriptomics approaches have identified significantly perturbed pathways and uncovered the causes of several diseases from a limited tissue-level perspective [81]. At the in vivo level, individual cells are subjected to a variety of environments, thus the diversity in individual cells far exceeds that identified by bulk omics. The development of single-cell technology has enabled profiling at the genomic, transcriptomic, and proteomic levels and provides tremendous opportunities to study cell-cell interactions and tumor heterogeneity [82]. Molecular features of tumors that bring the most heterogeneity, such as DNA, RNA, and proteins, can be captured through emerging single-cell genomics techniques. This technology has enabled researchers to gain a deeper understanding of the molecular composition of tumors, as well as to reveal unique insights into the mechanisms of tumor heterogeneity. By taking advantage of single-cell genomics techniques, researchers have been able to identify previously unknown tumor subtypes and develop novel therapeutic strategies to target them, which had the potential to improve the prognosis of colorectal cancer patients. For example, in a comparative analysis between bulk WES and single-cell WES, it was found that there was a significant underestimation of tissue heterogeneity compared to single cells [83]. In another study of the transition from ulcerative colitis to ulcerative colitis-associated colorectal cancer, the role of DPEP1, CD74, and CLCA1 in the progression of the disease was identified [84]. In another study, organoids developed from single cells showed a differential response to common drugs in closely related cells. This pointed to the inheritable biological states of individual cancers, which are due to a higher rate of molecular diversification in cancer cells [85].

Single-cell next-generation sequencing has been used to analyze tumor evolution and identify molecular variables associated with response to immunotherapy. In a study, two distinct Th-1 cell-like clusters were identified in a single T cell analysis of T cells derived from CRC patients. The CSCL13+ BHLHE40+ TH1- cell-like cluster was found to be associated with BHLHE40, while GXMK+ effector memory T cells were correlated with the expression of EOMES and RUNX3. Moreover, patients with microsatellite instability tumors were found to be associated with CSCL13+ BHLHE40+ TH1- cells. The enrichment of this subtype makes these patients more responsive to immune checkpoint blockade immunotherapies. In a single-cell study on the epigenetic level, it was found that DNA demethylation in cancer regions was correlated to histone modification in normal tissue [86].

Single-cell sequencing has uncovered the dynamic variations of the tumor microenvironment and immune system, which are critical for therapy resistance. In a study, the interaction between tumor-infiltrating lymphocytes and tumor cells was found to play a critical role in affecting the outcome of immunotherapy [87]. The dynamic role of CD8+ infiltrating T cells is known to be critical for the success of several immunotherapies. As more researchers and physicians embrace this technique, our understanding of the complexities of colorectal cancer in a broad context would be refined [88]

## 6. Liquid Biopsy

There have been significant strides made over the past few decades in the field of molecular profiling of cancer patients, but these require invasive tissue biopsies and lengthy timeframes to generate clinically relevant results. In contrast, approaches such as liquid biopsy has generated a lot of attention due to their non-invasive nature [89]. Liquid biopsy is similar to a blood test but can detect far more complex biomolecules from bodily fluids. It can detect several biomolecules including cell-free DNA, that have been significantly developed as a powerful tool to understand tumor heterogeneity in cancer patients. Cell-free DNA is constantly shed throughout the body, and in cancer patients, it becomes an easily accessible source of clinically actionable markers. The U.S. Food and Drug Administration (FDA) approved the use of epidermal growth factor receptor (EGFR) mutation testing from plasma as a companion diagnostic in 2016 to identify mutations [90]. This approach has several advantages such as being non-invasive and offering several advantages such as early detection and real-time monitoring of cancer and its treatment. It offers a better representation of tumor heterogeneity due to its circulatory origin that can capture biomarkers from multiple tis-sues. It has served as a tool to identify and characterize molecular alterations at several time points, which is nearly impossible to identify in a single conventional biopsy at a particular time [91,92]. In a large study of 1,397 CRC patients, it was identified that cfDNA sequencing identified most of the tissue-based biopsy sequencing characteristics [92].

These comprehensive profiling methods can be used to characterize several biomolecules, such as circulating tumor DNA and exosomes. Exosomes are small vesicles that contain a variety of biomolecules, including RNA, non-coding RNA (microRNA, lncRNA), proteins, and lipids [93]. This information can provide critical insights to complement existing regimens for treating cancer patients [93,94].

Circulating tumor ctDNA has become available for several cancers, offering several advantages, including ease of sampling, devising personalized treatment strategies, and monitoring response to therapies. Moreover, molecular characterization of circulating DNA has identified correlated genomic features that match with the corresponding tumors, thus having tremendous potential in clinical oncology [94]. Amplification of the ERBB2 gene is linked to a lack of response to anti-EGFR treatments [95]. ctDNA analysis using NGS has successfully identified this amplification in more than 96% of patients in the study. This is especially useful since archival tissue or biopsy is not required and this marker can be used as a surrogate in the form of ctDNA [96].

Another advantage of liquid biopsy is the ability to identify mutations associated with resistance and evaluate them to assess response to therapy [92]. Most importantly, resistance to ongoing treatment has emerged as a major challenge in clinical oncology. In a recent PROSPECT-C trial on metastatic colorectal cancer (CRC) patients, the aberrations in the RAS pathway and their association with resistance to anti-EGFR treatment in metastatic CRC were investigated. This study successfully showed that dynamics of resistance in both primary and acquired forms can be detected in both tissue and plasma. Interestingly, they found that almost 50% of metastatic CRC patients with wild-type KRAS and eligible for EGFR therapy had aberrations observed in RAS through cfDNA sequencing, thus not benefiting from anti-EGFR therapies [97]. In another study of 138 patients with gastrointestinal cancer, circulating tumor DNA (ctDNA) was found to be a more reliable indicator of response to therapies than standard clinical parameters [98]. Further, circulating tumor DNA was found to be an independent prognostic marker (HR 1.85, 95% CI 1.31–2.61) in a study on 1345 patients, [99]. In another study, post-operative circulating tumor DNA four weeks after surgery was associated with poor disease-free survival (HR 10.9, 95% CI 7.8–15.4). Also, adjuvant therapy was associated with improved outcomes in stage I, III, and IV patients [100]. Considering the significance of liquid biopsy, it has been proposed that the conventional Tumor extent (T), Lymph Node Invasion (N), and Metastasis (M) classification system can be strengthened by incorporating Liquid Biopsy (B) component (TNMB) [101]. The adoption of liquid biopsy in routine clinical colorectal cancer (CRC) care is hindered by several obstacles, including cost-effectiveness and the need for optimized protocols [102].

Apart from circulating tumor DNA, extracellular vesicles, containing DNA, RNA, proteins, and non-coding RNA, can be found in serum, saliva, and urine and has the potential to be used as markers for prognostic and predictive properties [103]. Plasma microRNAs, such as miR-20a, miR-24, miR-423-5p, miR-18a, miR-21, miR-29a, miR-92a, miR-106b, miR-133a, miR-143, and miR-145, have been found to have a di-agnostic utility in CRC [104]. Furthermore, several microRNAs are associated with metastasis. Interestingly, miR-106b-3p expression was found to be higher in patients with colorectal cancer (CRC) metastasis compared to early-stage tumors. At the molecular level, the miR-106b-3p target was identified to be DLC-1 [105].

**Figure 1:**
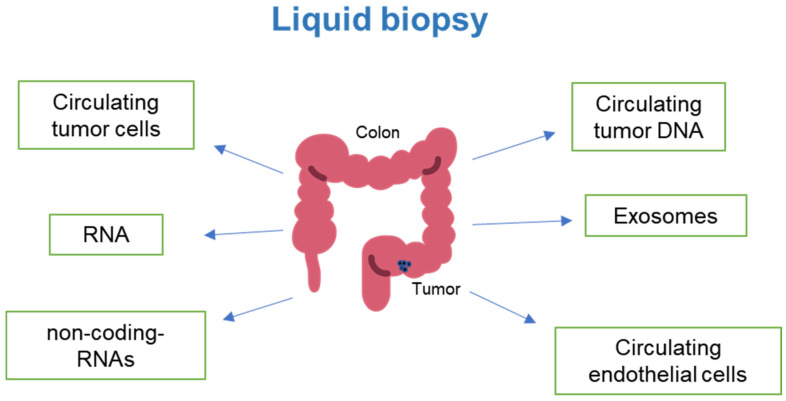
Emerging technologies are assisting in the quantification of circulating components in cancer patients, enabling the monitoring of disease progression and response to chemotherapy and immunotherapy (Image modified from: Flaticon.com).

The comprehensive evaluation of circular RNA, piRNA, tRNA, lncRNA, and snoRNA in the serum is emerging as an important addition to comprehensive molecular profiling of tumor patients pre- and post-therapy [106]. Interestingly, platelets, fragments of megakaryocytes, have emerged to play a role in detecting tumor progression and metastasis. Thrombocytosis has been linked to colorectal cancer (CRC) and elevated levels of interleukin-6 (IL-6) [107,108]. Furthermore, gene signatures related to platelets are enriched in hepatic metastasis of colorectal cancer. The specific genes identified were Fibrinogen beta (FGB) and von Willebrand factor (VWF) [109]. Platelets can also serve as an immune surveillance escape mechanism for circulating tumor cells, as they can activate them and trap them by forming thrombi [110]. Further understanding of circulating profiles using liquid biopsy will provide a powerful tool for designing personalized therapies and continually monitoring treatment efficacy in CRC patients (Figure 1).

## 7. Spatial Biology—Understanding Tumor Heterogeneity in CRC

Over the last few decades advances in genomics have significantly improved the diagnosis and treatment of CRC. These approaches have identified clinically actionable molecular alterations such as pathogenic mutation, amplification, insertion/deletion, and gene fusion. These genomic tools have started to shape the era of personalized therapies for CRC patients. However, despite their benefits, there are significant limitations as these technologies cannot fully capture the heterogeneity and complexity of CRC. 

One of several technologies that can strengthen this component is the inclusion of spatial biology. It can provide molecular-level variations in RNA and proteins and their distribution in healthy and diseased states. Additionally, it can help differentiate patients with cancer in the same stage or grade. Through the inclusion of spatial information, unprecedented resolution of the tumor microenvironment can be achieved, which in turn can significantly strengthen clinical and prognostic systems. This resolution includes the identification of novel cell and tissue level features, the complex roles of stromal and immune cells, cell-cell interaction, and invasion. These features can further enable the identification of novel subtypes of tumors that can be particularly useful for targeting cold tumors that are not vulnerable to immunotherapies. Identifying new subtypes of colorectal cancer (CRC) based on spatial biology can help clinicians manage CRC more effectively.

Recently, there has been an explosion of innovative spatial biology methods that can quantify the whole transcriptome and proteome across diverse tissues, such as multiple diseases and colorectal cancer (CRC). Spatial transcriptomics is based on three major principles: In laser capture microdissection (LCM) based methods, microscopy is used to identify a region and shape of interest. These sections are then selected and processed ex-situ. These methods have been applied to resolve heterogeneity, identify relatively pure cells, and perform differential analysis at the genomics, transcriptomics, and proteomics level [111]. Another method of in situ barcoding uses DNA barcodes on intact tissue samples and utilizes NGS-based methods, followed by computational algorithms to map expression information to spatial coordinates.

In solid-phase capture, tissues are sectioned onto a glass slide with a pre-arranged capture array of DNA oligonucleotide probes. The permeabilized RNA is reverse-transcribed to cDNA, which is subsequently sequenced using next-generation sequencing methodologies. The sequencing information obtained is then mapped to the aligned histology images acquired earlier. These technologies have generated large-scale atlases of various tissues, which have been instrumental in identifying new subtypes with unique cellular populations and their association with clinical outcomes [112]. Using selective barcoding, the Nanostring GeoMx Digit Profiler has been able to extract transcriptome and proteome profiles from FFPE tissues. It uses photo-cleavable oligonucleotide tags to attach to antibodies or RNA hybridization probes. Regions of interest are labeled using UV light, which releases light-sensitive linker multiplexed tags from the antibodies or RNA. These tags are collected through capillary action for Next-generation sequencing. Third, Imaging-based methods perform simultaneous acquisition of gene expression and spatial information through fluorescent imaging of tissue sections. Fluorescent in situ Hybridization (FISH) and In situ Sequencing (ISS) are two methods that quantify RNA molecules using optical microscopy. Recently, a new fluorescence imaging-based spatial platform has emerged. It uses a combinatorial fluorescence probe, imaging, and an AI-based decoding pipeline that can scale up to 60-plex with 12 different fluorophores [113].

Spatial technologies have provided a powerful tool for tumor profiling and hold significant potential for clinical oncology. Several studies have utilized this technology and have captured novel phenotypic features associated with colorectal cancer. In a recent spatial study, two types of cancer-associated fibroblasts (CAF-A and CAF-B) have been identified as playing a critical role in colorectal cancer [114]. CAFs have been shown to play an important role in the inhibition of NK cells and the disruption of cytokine and chemokine networks [115]. It was found that clusters enriched in CAFs had a decreased number of NK cells. These clusters also showed significant enrichment of macrophages. Tumor-associated macrophages are shown to be modulated by CAFs, thus affecting the activity of NK cells [116]. Furthermore, Cytokine-Activated Fibroblasts (CAFs) were found to be upregulated in patients receiving adjuvant chemotherapy [114]. In another study, spatial analysis was applied to better understand tissue remodeling due to tumorigenesis and assess molecular-level variations. They identified a reduction in the mesenchymal stem-like cell fibroblast subtype (NT5E+ subtype) along with an increase in cancer-associated FAP+ fibroblasts [117]. Further, this study identified metabolic perturbations resulting from FAP+ fibroblasts, including galactose, steroids, fatty acids, and non-essential amino acids. Along with other studies, this study identified an increase in macrophages (SPP1+ subtype) that are associated with poor progression-free survival in CRC patients [117,118]. Spatial studies have revealed a functional perturbation that is essential for uncovering the heterogeneity associated with colorectal cancer (CRC) pathogenesis and better understanding it. In a study of spatial variables associated with metastasis, the enrichment of immunosuppressive cells in the liver metastatic niche was identified. Immunosuppressive macrophages (MRC+, CCL18+) showed higher metabolic states in these niches and were found to be susceptible to anti-tumor treatments. Interestingly application of neo-adjuvant chemotherapy was found to be associated with a reduction in the metabolic activity of these phenotypes [119]. 

Spatial analysis has identified cellular pathways associated with tumorigenesis. It was found that endoplasmic stress activity was higher in the tumor region compared to adjacent normal tissue. Furthermore, there was an increased association of FOXP3 Treg cells, indicating a correlation between ER stress and immunosuppressive niches in the CRC microenvironment [120]. In another interesting study, spatial analysis was used to analyze host-bacterial interactions in the tumor microenvironment [121]. They identified that the presence of microbiota in the tumor microenvironment can significantly alter the tumorogenic trajectory, which is patient-specific. Further, these microbial communities can affect the response of patients to therapies and can affect their survival. The presence of *Fusobacterium* (F.) *nucleatum* is enriched in colorectal cancer (CRC) patients who experienced relapse after chemotherapy. The molecular mechanisms are influenced by toll-based receptors, autophagy, and microRNAs [122]. Moreover, bacteria have been found to metabolize chemotherapeutic drugs; for example, *Gammaproteobacteria* were found to metabolize gemcitabine [123]. Further, large-scale analysis of the TCGA dataset has revealed unique microbial signatures associated with distinctive cancers [124]. These and other similar studies have demonstrated the potential of spatial transcriptomics to advance our understanding of tumor biology and incorporate the knowledge for clinical benefit.

**Figure 2:**
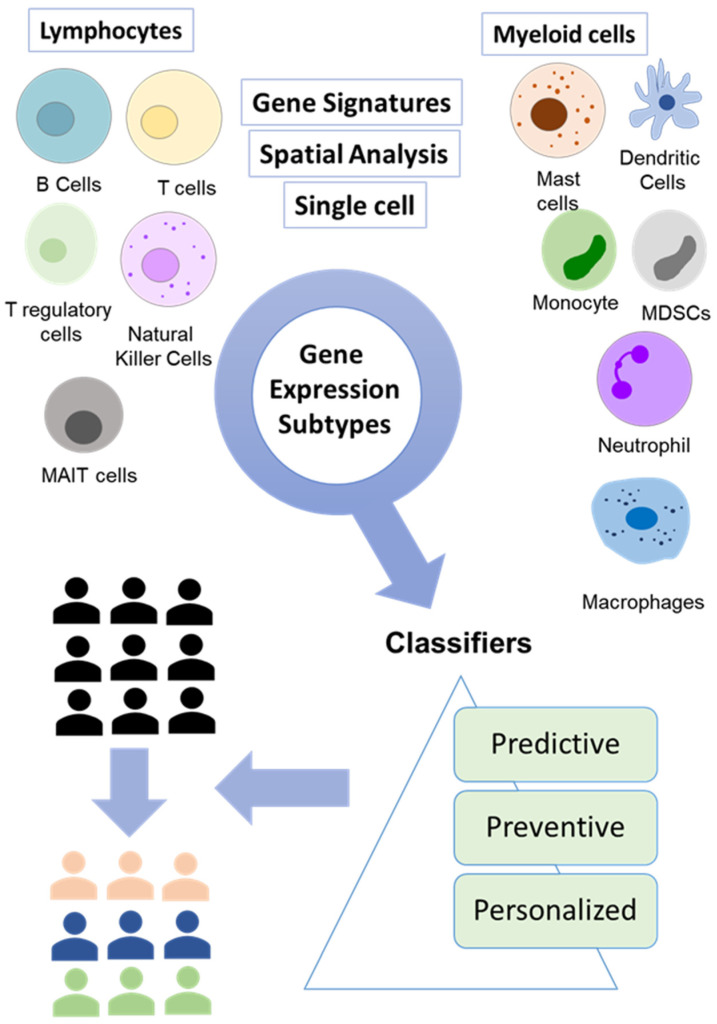
Integration of breakthrough technologies for accurate molecular profiling of tumors. A combination of gene signature, single-cell analysis, and spatial analysis is enabling the identification of new tumor subtypes that can be targeted using emerging therapeutics.

The understanding of hallmarks of cancer such as evasion of growth suppressors, genomic instability, dysregulated metabolism, and avoidance of immune response is shaping the newer ways through which we can better understand the complexity of cancer. Most recently, newer hallmark characteristics such as polymorphic microbes, epigenetic programming, phenotypic plasticity, and cellular senescence have added a layer of complexity [18]. At the same time, the intersection of these areas can lead to novel opportunities to exploit these mechanisms translationally. At the clinical level, this has added to the complexity of colorectal cancer as the variations in these factors can result in resistance or sensitivity to therapies such as chemotherapy or emerging therapies such as immunotherapies. Multiple advancements in liquid biopsy, gene signatures, higher-resolution single-cell analyses, spatial phenotyping, and data analysis have the potential to provide unprecedented access to the dynamics of cancer progression and resistance. However, there is a significant need to identify new markers with prognostic and predictive properties to strengthen personalized targeted therapies for individual patients. Moreover, the distribution of potentially therapeutically targetable biomolecules through spatial technologies has significantly advanced our understanding of underlying CRC heterogeneity. Additionally, single-cell sequencing has identified the highest resolution that can be applied to cancer patients to gain greater insights that can guide clinical decision-making. To identify these markers, it is essential to uncover the underlying molecular and biological mechanisms that drive tumorigenesis. Through the incorporation of multiple technologies, it is now possible to gain valuable insights into the immune dynamics of cancer patients that can significantly improve personalized treatment options for cancer patients (Table 2, Figure 2). These variables along with other molecular correlates can form a strong basis for the application of personalized therapies that can identify novel subgroups from an existing population that can be benefited from distinct therapies each targeting a specific subgroup. Additionally, the identification of novel immune cell subtypes and their interaction can have substantial clinical benefits as it can assist in the development of new therapeutic approaches that can modulate the activity of immune cells. For instance, the modulation of monocytes and tissue-associated macrophages to anti-tumor properties through emerging therapies. For example, novel drug delivery methods based on nanoparticles and approaches to modulate macrophages have shown promising results in controlling tumor growth [125,126]. Thus, the use of advanced technologies can not only assist in gaining deeper insights into patient heterogeneity but can also help in the development of novel intervention strategies for the management of CRC patients.

## 8. Challenges

Over the years, diverse approaches have been utilized for the diagnosis and treatment of cancer, with the most commonly used being TNM staging. More recently, transcriptome-based comprehensive evaluation has emerged as a promising tool for the clinical evaluation of tumors. Despite significant advancements in the clinical management of CRC using diverse approaches such as TNM staging and comprehensive assessment of molecular alteration, there are still several limitations due to the heterogeneity of CRC leading to a lack of accuracy, reproducibility, and limited ability to capture treatment response. It is therefore essential to incorporate newer technologies such as novel gene signature-based methods, single cell, and spatial biology in the routine clinical care of CRC patients. However, successful integration of these technologies requires overcoming several technical hurdles. Each technology provides a unique hurdle that needs to be overcome to fully realize its potential of precision medicine [152,153]. Some of these challenges are briefly discussed below.

The quantification of biomolecules in tumors, their microenvironment, and circulation has greatly facilitated the identification of biomarkers for various diseases, including colorectal cancer. Our understanding of complex diseases has been transformed through the incorporation of several techniques ranging from low-throughput qPCR to high-throughput RNA-seq. Recently, single-cell genomics has provided a higher resolution of tissue dynamics. However, a significant drawback of this approach is the loss of 3D or spatial context of the biomolecules, due to the disassociation of cells before analysis. Further, since sRNA-seq and its associated dissociation step can lead to gene expression induction, this can lead to the misidentification of cell populations. For example, a study on muscle tissue found that dissociation of normal muscle tissue can induce an expressional signature that is also activated during muscle injury, thus contaminating the normal tissue with an injury-related gene signature [154]. Further other studies have also reported that the dissociation of cells for single cell sequencing protocols can trigger stress responses, leading to the induction of stress-related genes resulting in inaccurate results [155,156]. Therefore, it is essential to resolve these sources of variations so that accurate and precise information that can be utilized in patient care.

Another technology, spatial transcriptomics has recently emerged as a tool for identifying potential therapeutic targets. This has important implications for clinical oncology, as it has started to provide clinically relevant prognostic and personalized information [157]. Improved spatial maps of diseased networks are likely to identify in-situ interactions of tumor cells with adjacent tissue and immune cells, providing a window of opportunity to unravel interacting biological networks and opportunity to therapeutically target them. Several challenges exist with current spatial genomic approaches, such as depth, resolution, sensitivity, and limited tissue size. Additionally, there are computational challenges associated with tissue segmentation and deconvolution of cellular populations in a tissue. Increased effort in the field of computational algorithms will help to maximize the benefits of spatial technologies [158]. As larger studies become available, the prognostic and predictive power, as well as its sensitivity, is expected to increase. To fully realize the potential of personalized medicine, it is therefore critical to improve, refine, and validate the findings of these technologies before their integration into routine clinical practice. Along with the development of these tools, it is essential to optimize the computational algorithms and data analysis tools for a seamless flow of information from the laboratory to clinical settings. Additionally, it is essential to expand the personalized perspective of medicine by incorporating an equitable component to healthcare to ensure that patient-centric personalized medicine is accessible to everyone.

Apart from advances in transcriptomics, the field of metabolomics holds significant potential to revolutionize our understanding of cancer and prognosis [159]. Cancer cell metabolism is a central component of tumorigenesis as it involves sequestering essential nutrients from a nutrient-poor tumor microenvironment [160]. Metabolic reprogramming has emerged as a critical mediator of cancer progression and has the potential for clinical utility [161]. The rapid development of analytical techniques such as mass spectrometry, chromatography, X-ray crystallography, and nuclear magnetic resonance (NMR) has led to the emergence of metabolomics, which has become an important field along with genomics, transcriptomics, and proteomics. Metabolomics is essential for generating a complete biological information profile of a given sample [162]. Several studies have profiled metabolites as a result of metabolic programming in tumor tissues [163,164,165]. In an interesting study on FFPE and fresh CRC samples, Arima et. al. identified perturbations in metabolic profiles and functioning of mitochondria of colorectal cancer compared to normal colon [161]. the authors identified perturbed cellular metabolism and a reduction in the levels of alpha-ketoglutarate which plays a central role in the tricarboxylic acid cycle. Additionally, the study also showed perturbations in amino acids in tumor tissue. The branched chain amino acids such as isoleucine and leucine were found to be elevated in tumor tissues. As these amino acids are directly degraded to acetyl-CoA and succinyl-CoA, their elevated levels point to mitochondrial dysfunction in cancer cells through the transfer of protons (H+) to metabolic matric water. The elevated levels of degraded metabolites of BCAA in the form of acetyl-CoA and succinyl-CoA, point to mitochondrial dysfunction in cancer cells. This dysfunction involves multiple molecular factors, including impaired proton transfer to metabolic matrix water, leading to less deuterium content. Healthy mitochondria function involves the fundamental function of inhibiting deuterium oncoisotope accumulation inside the healthy cell. The continuous accumulation of deuterium leads to cancer development and holds promise as a prognostic biomarker in multiple cancer studies [166,167]. Further advances in the field of metabolomics can provide critical insights that can not only be used as biomarkers for prognostic significance but also therapeutic significance. These metabolites can be used to quantify individual differences in tumor drug metabolism, monitor the efficacy of drugs, and predict resistance to drugs [164]. These advances hold significant promise in increasing the efficacy of precision medicine in colorectal cancer.

In the last few years, technological developments in the healthcare field have been rapid and are continuously evolving [168,169]. One of the most revolutionizing breakthroughs was the introduction of the IoT (Internet of Things) concept within the surgical practice [169]. Additionally, wearable devices, implants, and ingestible sensors can monitor pH levels, gut health, temperature, blood, enzyme, and microbiome composition which can enable early detection of colorectal cancer [170]. Further, artificial intelligence (AI) has started to improve the analysis of colonoscopic images providing accurate detection of early-stage polyps, which can lead to early intervention and improved outcomes [171]. Miniaturized devices hold significant potential for real-time monitoring of molecular variables in individuals, offering a timely window of opportunity for applying personalized medicine and precision targeting of colorectal cancer.

## 9. Conclusions

The advancements in genomics have significantly improved patient care yet its full potential is yet to be realized. While chemotherapy, targeted therapy, and immunotherapy have shown success, lack of responsiveness and therapy resistance remains a challenge for a significant number of patients. Although the TNM staging system has widespread application but has its significant challenges. There is a need to incorporate novel innovative approaches that can assist the current TNM staging system. One such approach is the clinical expansion of comprehensive genomic profiling that has advanced the area of personalized medicine. Gene signatures can fill in some of the gaps, particularly through the provision of equitable medicine. Breakthroughs in transcriptomics, spatial and liquid biopsy can provide unprecedented access and resolution to the patient’s health state. Further, the identification of novel immune subtypes has provided an enhanced viewpoint of diverse immune landscapes in patients that can be therapeutically targeted. The role of previously unknown immune cell subtypes has shown their importance at the clinical level and holds great promise in precision oncology. Further, these tools can assist in the identification of molecular signatures in non-responders, potentially reducing the toxic side effects and financial burden on patients. These technologies have the potential to integrate with the existing clinical management pipeline and provide more comprehensive treatment options for CRC patients. Further, the incorporation of these parameters can strengthen the core pillars of predictive, preventive, and personalized medicine which are highly dependent on interdisciplinary, patient-specific therapeutic interventions. Therefore, the continued collaboration between healthcare providers, researchers, and policymakers to integrate these diverse approaches will facilitate the better management of CRC.

## Figures and Tables

**Table 1:** Recent prognostic gene signatures in colorectal cancer.

Gene-Signature	Genes	Patient Outcome	Reference
DNA repair-related gene signature	11-gene signature comprising of *ARPC1B*, *BCL2*, *CDA*, *ERBB2*, *FUT4*, *NPR2*, *PLD6*, *POLR2B*, *PSME2*, *RAD1*, and *UBE2D2*.	Disease-free survival, H.R = 2.40, 95% C.I = 1.67–3.44; *p* < 0.001.	[63]
8 gene-signature	8 gene signatures comprising *ATOH1*, *CACNB1*, *CEBPA*, *EPPHB2*, *HIST1H2BJ*, *INHBB*, *LYPD6*, and *ZBED3*.	Overall survival, HR = 1.39, 95% CI = 1.24 to 1.56.	[64]
Hypoxia signature	12-gene signature comprising of *TNFAIP8*, *ORAI3*, *MINPP1*, *MBTD1*, *TRAF3*, *CYB5R3*, *ZBTB44i CASP6*, *DTX3L*, *FAM117B*, *PRELID2*, and *IRF1*.	Worse prognosis in patients with adjuvant chemotherapy, H.R = 5.1, 95% C.I = 2.51–10.35; *p* = 0.001.	[65]
Recurrence-associated	6-gene signature comprising of *COX6A1*, *ERN1*, *IFITM2*, *S100P*, *STK24* and *TMTC3*.	Predictor of recurrence, H.R = 3.40, 95% C.I = 1.76–6.56; *p* < 0.001.	[66]
lncRNA signature	A 6-gene signature comprising of SNHG16, AL590483.1, ZEB1-AS1, AC107375.1, AC068580.3, and AC147067.1/	Overall survival, H.R = 1.21, 95% C.I = 1.14–1.301; *p* < 0.001.	[67]
chemotherapy-resistant gene signature	A 4-gene signature comprising of *CD22*, *CASP1*, *CISH*, and *ALCAM*.	Oxaliplatin resistance, H.R = 2.77, 95% C.I = 2.03–3.78; *p* < 0.001.	[68]
Ferroptosis	A 20-gene signature composed of *ANGPTL7*, *CDKN2A*, *FADS2*, *GCH1*, *GDF15*, *IL6*, *LINC00472*, *MAPK3*, *NNMT*, *NOX4*, *PTGS2*, *RGS4*, *SCD*, *SLC1A4*, *SLC2A3*, *SOCS1*, *TAZ*, *TF*, *TP63*, and *VLDLR*.	Overall survival, HR: 2.11, 95% CI: 1.40–3.17, *p* < 0.001.	[69]
fibroblast-related gene signature	A 11-gene signature composed of *POLR2B*, *GAS6*, *CRY1*, *BCL2L1*, *ARG1*, *ORA13*, *TRAF3*, *ZSWIM4*, *IRF1*, *LEMD1*, and *ACTB*.	Adjuvant therapy, HR = 3.63, 95% CI 2.24–5.88, *p* < 0.001.	[70]
Metabolism	An 18-gene signature composed of *LIPG*, *PSME1*, *METTL2B*, *DDX52*, *CS*, *NHP2*, *POMT1*, *OGDHL*, *AMACR*, *ALOX12B*, *ACOX2*, *RPS25*, *CYP2D6*, *PLA2G4D*, *INHBB*, *NPR2*, *PLCE1*, *LIPG*, and *ABCD4*.	Overall survival, H.R = 2.12, 95% C.I = 1.67–3.44; *p* < 0.001.	[71]
Metabolism	A 10-gene signature composed of *CD163L1*, *FAM13B*, *HDAC6*, *HPR*, *NR2C2*, *RAB12*, *SIRT2*, *TBC1D14*, *TLK2*, and *TBC1D12*.	Disease-free survival, H.R = 2.76, 95% C.I = 1.56–4.82; *p* < 0.001.	[72]
Immune-associated gene signature	A 4-gene signature composed of *TGFB1*, *PTK2*, *RORC*, and *SOCS1*.	Overall survival, H.R = 1.76, 95% C.I = 1.05–2.95; *p* < 0.02.	[73]
20-gene signature	20-gene signature composed of The genes involved are *ANGPTL4*, *BAFT3*, *CCL18*, *CD36*, *HAVCR2*, *IL6*, *ITGAM*, *MS4A4A*, *NFATC2*, *NGFR*, *OLFML2B*, *SFRP1*, *SNAI1*, *THBD*, *TREM2*, *CLCA4*, *CXCL5*, *MMP1*, *PIAS4*, and *WNT5A*.	Overall survival, (H.R = 2.32, 95% C.I = 1.69–3.19; *p* < 0.001.	[74]
EMT gene signature	6 gene signature composed of *BP2*, *MAPT*, *BIRC5*, *PLXNA1*, *CHGA*, and *SPP1*.	H.R = 5.07, 95% C.I = 3.05–8.43; *p* < 0.001.	[75]
67-gene signature	CINSARC score differentiated patients based on overall survival.	Overall survival, H.R = 2.45, 95% C.I = 1.31–4.59; *p* < 0.001.	[76]
Lipid metabolism gene signature	Glycerolipid gene signature differentiated CRC patients based on survival.	H.R = 0.63, 95% C.I = 0.42 - 0.94; *p* < 0.001.	[77]
lncRNA signature	A lncRNA signature composed of LINC01116, AC005838.2, SH3PXD2A-AS1, VIMS-AS1, SH3BP5-AS1, AC092279.1, AC026355.1, AC027020.2, and LINC00996.	Overall survival, H.R = 1.17, 95% C.I = 1.10–1.24; *p* < 0.001.	[78]
Cupropotsis-related gene signature	CupRLSig gene signature.	Overall survival, H.R = 1.162, 95% C.I = 1.06–1.27; *p* < 0.001.	[79]
collagen signature	16 collagen features using 327 stage I–II CRC patients showed lower Immunocore.	AUC of 0.925 (training cohort, 95% CI: 0.895–0.956) and 0.911 (validation cohort, 95% CI: 0.872–0.949)	[80]

**Table 2:** The emerging roles of immune cells and their subtypes.

Immune Cell	Clinical Utility of Cellular Subtypes	References
B cells	B lymphocytes play a dual role in the tumor microenvironment and are dependent on the stage, location, and grade of the colorectal tumor. Several subtypes of B cells have been identified. In CRC, single-cell sequencing has identified 5 distinct subtypes of B cells with differential distribution in the tumor-inflamed subgroup.	[127,128,129]
Dendritic cells	Dendritic cells play a central role in the immune response against colorectal cancer through the presentation of tumor antigens to the T cells, but these are prone to tumor-mediated immunosuppression. Several new subtypes of dendritic cells have been characterized. Single cell analysis of metastatic colorectal cancer samples has identified distinct subpopulation of dendritic cells (DC3) and SPP1 macrophages associated with liver metastasis.	[130,131,132]
Monocytes	Monocytes perform several functions that include phagocytosis, mediation of anti-tumor immunity, and remodeling of the extra-cellular matrix. Monocyte subsets with different transcriptomic and functional properties have been identified. FCN1+ monocyte-like cells have shown to lead to the formation of C1QC+ and SPP1+ TAMs in colorectal cancer.	[133,134,135]
Macrophages	Macrophages exhibit a range of functions, spanning from angiogenesis and metastasis to cytotoxic tumor-killing activities. Immunosuppressive interaction clusters of cells, including tumor-associated macrophages (TAMs) and cancer-associated fibroblasts (CAFs), contribute to immune evasion in the tumor microenvironment. Two distinct TAMs have been identified: C1QC+ TAMs (pro-inflammatory, enrichment of inflammation) and SPP1+ TAMs (anti-inflammatory roles in CRC).	[135,136,137]
Mast cells	Mast cells can alter the tumor microenvironment milieu through the secretion of cytokines, chemokine, and other mediators. Mast cells display distinct gene expression patterns based on amino acid metabolism as identified through gene expression deconvolution analysis. In a recent study, density of mas cells was found to be lower in CRC but with a shift from resting cells (CMA1^high^) to activated state (TPSAB1^high^, CPA3^high^, and KIT^high^).	[138,139,140]
Neutrophils	Neutrophils can directly contribute to anti-tumor immunity through Antibody-dependent cellular cytotoxicity. Neutrophil-enriched subtypes were found to correlate with pro-inflammatory subtypes in colorectal cancer. Single cell RNA sequencing has identified novel subsets of neutrophils that are present in circulation in cancer	[141,142,143]
Natural killer cells	NK cells possess cell lysing anti-tumor properties, but they are prone to resistance in the tumor microenvironment. Recent studies have identified novel subtypes of Natural Killer cells that may have prognostic and predictive value. In a recent study, three distinct NK cell subtypes have been identified in colorectal cancer.	[143,144,145]
T cells	Differential expression of genes in activated, dysfunctional or exhausted T cells can assist in the identification of novel subtypes that can be exploited clinically. In a recent study, CD8+ T cells subpopulations with distinct properties such as tumor-reactive signaling modules and IFN-γ signaling with particularly identification of ‘pseudo-hot’ tumors characterized by inflammation but lack of significant CD8+ T cell infiltration.	[146,147]
T regulatory cells	T reg cells are involved in the maintenance of self-tolerance. Single-cell analysis revealed distinct T-regulatory cells with opposite clinical outcomes.	[148]
MDSCs	Different types of MDSCs such as M-MDSCs, PMN-MDSCs, e-MDSCs, and F-MDSCs can confer differential survival determination of prognostic properties.	[149]
MAIT cells	MAIT cells are innate-like T cells that identify small molecules of antigenic origin. These cells have exhibited overlapping transcriptional profiles with CD4 T cells and FOXP3 cells.	[150,151]
